# Meeting of the American Medical Association, at Cincinnati

**Published:** 1850-06

**Authors:** 


MEETING OF THE AMERICAN MEDICAL ASSOCIATION, AT CINCINNATI.

This was one of the largest assemblages of delegates, that the objects of the Association have called together. It was perhaps excelled by the meeting in Boston.

Among the earliest duties of the Association was the election of officers. The Association confirmed the selections of the committee, appointed for this purpose. The following are the names of the officers, for the ensuing year:

*President.*—Dr. R. D. Mussey, of Cincinnati.

*Vice Presidents.*—Dr. J. B. Johnson, of Missouri, 1st; Dr. A. Lopez, of Alabama, 2d; Dr. D. Brainard, of Illinois, 3d; and Dr. G. W. Norris, of Pennsylvania, 4th.

*Secretaries.*—Dr. Alfred Stillé, of Pennsylvania; Dr. H. Desaussure, of South Carolina.

*Treasurer.*—Dr. Isaac Hays, of Pennsylvania.

We must reserve our remarks on the proceedings of the Association, for another number.

